# The implications of lag times between nitrate leaching losses and riverine loads for water quality policy

**DOI:** 10.1038/s41598-021-95302-1

**Published:** 2021-08-12

**Authors:** R. W. McDowell, Z. P. Simpson, A. G. Ausseil, Z. Etheridge, R. Law

**Affiliations:** 1 AgResearch, Lincoln Science Centre, Lincoln, New Zealand; 2 Department of Soil and Physical Sciences, Lincoln University, Lincoln, New Zealand; 3 Manaaki Whenua Landcare Research, 17 Whitmore St, Wellington, New Zealand; 4 Kōmanawa Solutions Ltd, Christchurch, New Zealand; 5 Manaaki Whenua Landcare Research, Manawatu Mail Centre, Private Bag 11052, Palmerston North, New Zealand

**Keywords:** Biogeochemistry, Hydrology, Environmental sciences, Environmental impact

## Abstract

Understanding the lag time between land management and impacts on riverine nitrate–nitrogen (N) loads is critical to understand when action to mitigate nitrate–N leaching losses from the soil profile may start improving water quality. These lags occur due to leaching of nitrate–N through the subsurface (soil and groundwater). Actions to mitigate nitrate–N losses have been mandated in New Zealand policy to start showing improvements in water quality within five years. We estimated annual rates of nitrate–N leaching and annual nitrate–N loads for 77 river catchments from 1990 to 2018. Lag times between these losses and riverine loads were determined for 34 catchments but could not be determined in other catchments because they exhibited little change in nitrate–N leaching losses or loads. Lag times varied from 1 to 12 years according to factors like catchment size (Strahler stream order and altitude) and slope. For eight catchments where additional isotope and modelling data were available, the mean transit time for surface water at baseflow to pass through the catchment was on average 2.1 years less than, and never greater than, the mean lag time for nitrate–N, inferring our lag time estimates were robust. The median lag time for nitrate–N across the 34 catchments was 4.5 years, meaning that nearly half of these catchments wouldn’t exhibit decreases in nitrate–N because of practice change within the five years outlined in policy.

The restoration of good water quality requires remedial action to prevent the loss of contaminants like nitrate-nitrogen (N) from land to water. The leaching and transport of nitrate–N to waterbodies can take days, months, years, or decades depending on biogeochemical processes like attenuation, flow-paths and flow rates^1,2^. The time taken for changes in nitrate–N leaching from the root zone and soil profile to be reflected in the nitrate–N load delivered to a waterbody is termed ‘lag time’^1^. Despite action to mitigate nitrate–N leaching from agricultural land, long lag times for past N inputs which are already in transit through the subsurface can sustain or even increase future nitrate loads in rivers^2^. Consequently, understanding the magnitude of lag times within and between catchments is essential in knowing which actions will be most effective and where and when they should be implemented^3^. Remaining ignorant of these effects can raise expectations that water quality will improve within unrealistic time frames. This could compromise the ability to meet policy objectives. For instance, policy in Europe and New Zealand has set objectives to improve water quality 5 or 10 years^4,5^. However, not accounting for lag times longer than 5 or 10 years may lead to unnecessary regulatory change and erode the confidence of landowners to invest in mitigation actions.

Determining lag times is difficult, often hindered by infrequent data sampled over short time frames^6^. Much more is known for deep groundwater, with typical lags up to decades^7^, but often the greatest nitrate contributions are associated with shallow groundwater or surface runoff pathways^8,9^, with lags ranging from about 1 to 10 years^10^. Disentangling flow paths requires extensive data and modelling which are both time consuming and expensive^10,11^. However, some simple statistical approaches exist to approximate average lag times. For example, one technique called cross-correlation compares the two time-series (e.g., an input and output) to determine how much of a delay occurs before the shape of the input best matches the shape of the output. This technique has been used to empirically approximate lag times for total N and phosphorus inputs as losses from the land and outputs as concentrations or loads in the river^12^.

Owing to the requirement for long-term datasets, modelling lag times has been limited to relatively few case studies^13^. Studies at the national scale are rare but owing to their size and geographic representativeness are helpful in elucidating the influence of catchment characteristics or climatic variation on lag times.

Our aim was to calculate the magnitude of lag-times between annual rates of nitrate–N leaching from land and nitrate–N loads in receiving rivers in New Zealand. We use data from the National River Water Quality Network. The Network drains approximately 50% of New Zealand’s land area and exhibits a broad range of climates, slopes, geologies, and land uses (Table 1) that vary from catchments with a large proportion of intensive agriculture to catchments under minimally disturbed conditions (MDC), i.e. reference state^14^. After calculating lag times, we use these data to provide a commentary on their implications for policy to improve water quality. We use recently announced policy for water quality improvement in New Zealand^4^ as an example.

**Table 1:** Summary statistics for biophysical parameters describing the 77 catchments of the National River Water Quality Network.

Variable	Mean	SEM^1^	Minimum	Quartile 1	Median	Quartile 3	Maximum	Data source
Area (km^2^)	2640	423	15	435	1130	3229	20,540	^75^
Population	13,393	4403	3	240	2513	7227	297,098	^76^
Mean slope (degrees)	4.2	0.3	0.7	2.3	3.3	6.1	12.6	^75^
Mean altitude (m)	621	33.0	63	370	638	815	1424	^75^
Mean monthly rainfall (mm)	148	4.8	69	126	139	160	308	^75^
Mean monthly runoff (mm)	77	4.6	5	55	69	90	228	^77^
Baseflow fraction	0.36	0.02	0.03	0.19	0.29	0.48	0.88	^2^
Olsen P (mg kg^−1^)	32	0.8	23	27	32	36	60	^78^
Intensive agricultural land (%)	41.7	3.6	0	14.0	34.8	66.8	99.9	^79^
Forest (%)	53.7	3.7	0.1	24.8	57.6	81.2	100.0	^79^
Rangeland (%)	4.4	1.0	0	0.3	1.2	3.8	52.2	^79^
Strahler stream order	5	0.2	2	5	6	6	8	^80^
Potential monthly evapotranspiration (mm)	51.2	0.5	39.6	47.3	51.5	54.9	61.4	^81^

Geology was added as a categorical variable assigned to either sedimentary, metamorphic or volcanic^74^.

^1^*SEM* standard error of the mean.

^2^Calculated according to the method of Pelletier and Andréassian^55^.

## Results

### Nitrate leaching losses and catchment loads

**Figure 1:**
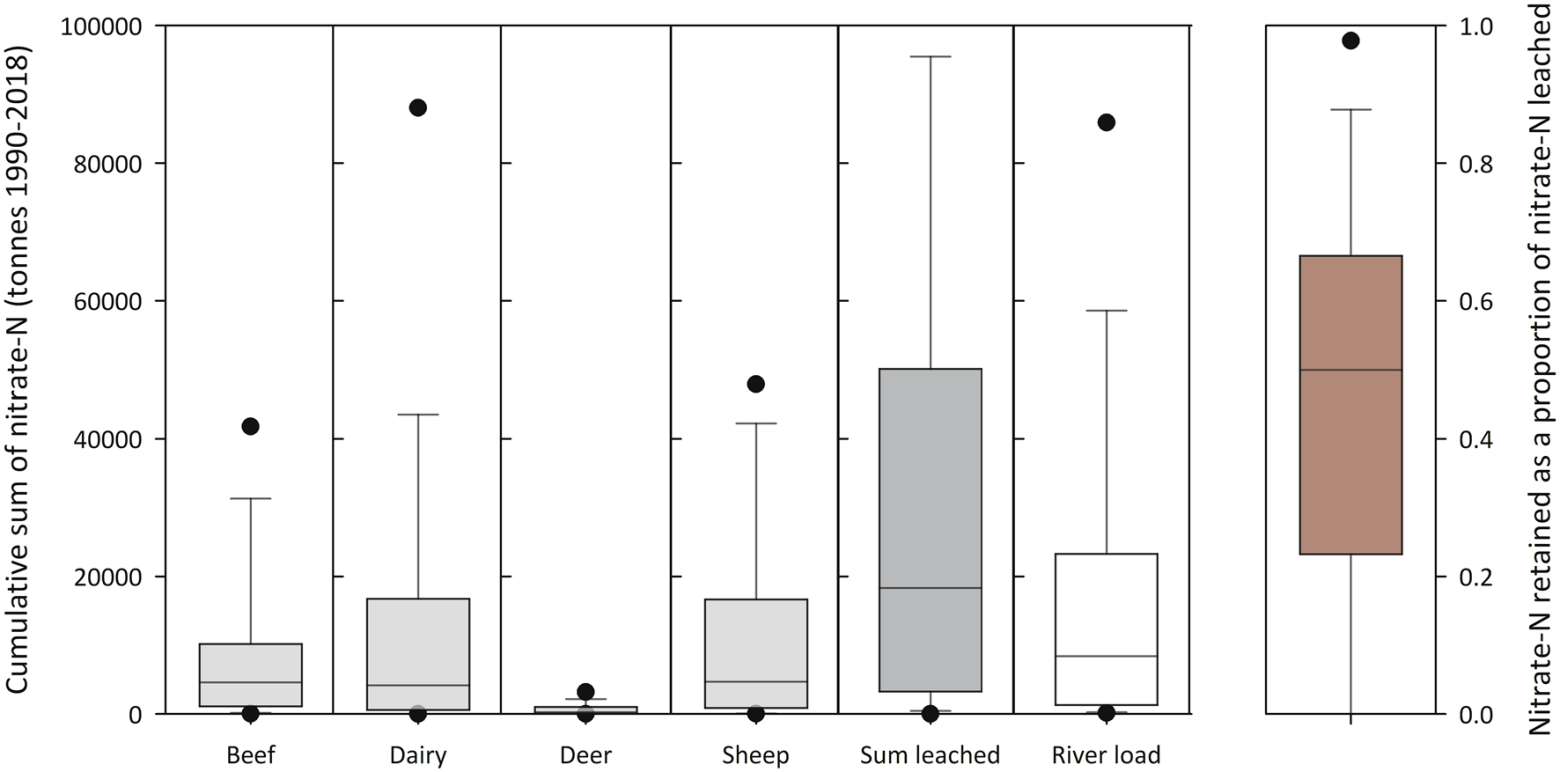
Range of the cumulative sum of nitrate–N leached (tonnes) over 1990–2018 for each livestock class, the sum of livestock classes and the load in the river. Also given is the range of nitrate–N loads retained in the river relative to the sum leached. Boxes describe the 25th, 50th and 75th percentiles, whiskers are the 5 and 95th percentiles. Outliers are indicated by black dots.

Over the period studied (1990–2017), nitrate–N leaching losses from the catchments totalled 3,641,415 tonnes (Supplementary Table S1). This compared to 6,453,000 tonnes of N that had been applied largely as urea from 1990–2015^15^ to agricultural land (> 95% pastoral farming) across New Zealand. The ratio of N applied to N leached compares well to the aggregate catchment area of the Network which covers approximately 50% of New Zealand land area^14^. Of the land uses considered, dairying accounted for the majority of nitrate–N leached (1,913,484 tonnes) while sheep and beef land and deer-farmed land contributed 1,642,909 and 56,925 tonnes, respectively (Fig. 1).

Catchment nitrate–N load ranged from 27 to 20,569 tonnes and generally reflected catchment size (*r* = 0.51), but yield, ranging from < 0.1 to 13.9 kg N ha^−1^ yr^−1^, did not (*r* = − 0.13; Table 2). This difference reflects variation caused by other factors such as agricultural area and livestock numbers between the catchments (Supplementary Table S1). Unsurprisingly, the median annual yield and load from the MDC catchments (2 kg N ha^−1^ yr^−1^; 1,336 tonnes yr^−1^) was lower (*P* < 0.05; Mann–Whitney test) than those with intensive agricultural activity (10 kg N ha^−1^ yr^−1^; 12,494 tonnes yr^−1^).

**Table 2:** Mean yield (± standard error) of nitrate–N and lag times (years) calculated using cross-correlation (using pre-whitened data) and cumulative generalised additive models (GAMs) and the resulting mean lag time after filtering out sites that were either impacted by hydroelectric schemes or were recently disturbed causing uncharacteristically high sediment loads, or under minimally disturbed conditions (MDC), i.e. > 90% native forest, mountains or scrub.

Code	Mean yield (kg N ha^−1^ yr^−1^)	Lag time			Mean transit time	References
		Cross correlation	Cumulative GAM	Filtered mean		
AK1	18.4 ± 1	(1.5)	(9)	Impacted	–	
AK2	7 ± 0.4	1.0	3.5	2.3	–	
AX1	0.9 ± 0.1	(5.7)	(4.5)	MDC	–	
AX2	0.8 ± 0.1	–	(9.5)	MDC	–	
AX3	0.4 ± 0.1	(3.0)	–	MDC	–	
AX4	1.4 ± 0.1	–	–	–	–	
CH1	0.9 ± 0.1	(5.8)	–	MDC	–	
CH2	5.6 ± 0.2	3.5	5	4.3	–	
CH3	1.2 ± 0.1	–	–	MDC	–	
CH4	3.9 ± 0.3	–	4	4.0	3.7	^31^
DN1	3.3 ± 0.1	1.0	5.5	3.3	–	
DN10	–	–	–	MDC	< 1	
DN2	2.9 ± 0.1	5.0	7.5	6.3	–	
DN3	3.5 ± 0.1	–	–	–	–	
DN4	2.7 ± 0.1	(5.7)	–	Impacted	–	
DN5	6.3 ± 0.1	1.0	3	2.0	–	
DN6	2.5 ± 0.1	–	–	–	< 0.5	^82^
DN7	3.2 ± 0.1	–	5.5	5.5	< 0.5	^82^
DN8	7.2 ± 0.3	–	4	4.0	1–2	^83^
DN9	1.7 ± 0.1	3.0	–	–	< 1	^83^
GS1	7.5 ± 0.2	6.0	–	6.0	–	
GS2	9.7 ± 0.2	(1.5)	(9.5)	Impacted	–	
GS3	9.9 ± 0.2	–	–	–	–	
GS4	4.2 ± 0.1	–	–	–	–	
GY1	3.1 ± 0.1	(6.0)	–	Impacted	–	
GY2	6.3 ± 0.2	–	4.5	4.5	–	
GY3	0.6 ± 0.1	(3.5)	–	MDC	–	
GY4	–	–	–	MDC	–	
HM1	15.4 ± 0.4	–	–	MDC	–	
HM2	29.7 ± 0.3	–	4.5	4.5	–	
HM3	17.2 ± 0.2	1.5	3.5	2.5	–	
HM4	21.1 ± 0.2	–	7	7.0	–	
HM5	27 ± 0.5	–	7.5	7.5	–	
HM6	22.4 ± 0.4	–	–	–	–	
HV1	2.2 ± 0.1	12.3	6.5	9.4	–	
HV2	11.2 ± 0.2	–	–	–	4–6	^84^
HV3	6.5 ± 0.1	12.0	–	12.0	4–6	^84^
HV4	1.0 ± 0.1	(4.7)	–	MDC	0.5–2	^84^
HV5	3.1 ± 0.1	–	–	–	–	
HV6	3.6 ± 0.1	–	–	–	–	
NN1	2.5 ± 0.1	–	–	–	–	
NN2	–	–	–	MDC	–	
NN3	0.4 ± 0.1	(2.5)	–	MDC	–	
NN4	2.2 ± 0.1	1.5	4	2.8	–	
NN5	2.1 ± 0.1	–	–	MDC	–	
RO1	2.3 ± 0.1	–	3	3.0	–	
RO2	5.5 ± 0.1	1.0	–	1.0	–	
RO3	6.6 ± 0.2	–	–	–	–	
RO4	1.1 ± 0.1	–	(6.5)	MDC	–	
RO5	6.6 ± 0.1	–	–	–	–	
RO6	3.4 ± 0.1	3.5	6.5	5.0	–	
TK1	7 ± 0.2	6.5	7.5	7.0	–	
TK2	7.9 ± 0.2	0.5	3	1.8	–	
TK3	3.5 ± 0.1	1.5	6.5	4.0	–	
TK4	1.1 ± 0.1	–	(4)	MDC	–	
TK5	15.1 ± 0.4	–	–	–	–	
TK6	2 ± 0.1	5.6	–	5.6	–	
TU1	6.6 ± 0.1	(1.0)	–	Impacted	–	
TU2	1.4 ± 0.1	–	–	MDC	–	
WA1	17.2 ± 0.2	–	–	–	–	
WA2	18.8 ± 0.2	–	7.5	7.5	–	
WA3	49.7 ± 0.5	–	4.5	4.5	–	
WA4	6.1 ± 0.1	–	6	6.0	6–10	^85^
WA5	7.3 ± 0.2	–	8	8.0	3–6	^85^
WA6	9.2 ± 0.2	–	6.5	6.5	3–6	^85^
WA7	13.7 ± 0.1	–	9.5	9.5	9–11	^85^
WA8	13.6 ± 0.1	–	–	–	0.5–5	^85^
WA9	14 ± 0.1	–	–	–	0.5–5	^85^
WH1	0.6 ± 0.1	–	(5.5)	MDC	–	
WH2	16.9 ± 0.3	–	–	–	–	
WH3	8 ± 0.1	–	2.5	2.5	–	
WH4	17.2 ± 0.2	–	3.5	3.5	–	
WN1	2.1 ± 0.1	–	–	–	–	
WN2	–	–	–	MDC	–	
WN3	9.8 ± 0.2	–	–	–	–	
WN4	10.2 ± 0.2	–	5.5	5.5	–	
WN5	0.6 ± 0.1	–	–	MDC	–	

The mean hydrological transit time for baseflow is also given for reference. Lag times in parentheses were for sites that were either impacted or under MDC and excluded from further analysis.

^1^A lag time either could not be calculated or no data exists.

We calculated the nitrate–N output as river load relative to the nitrate–N input via nitrate–N leaching and recorded the difference as the percentage of nitrate–N retained by the catchment. Nitrate–N retained across the catchments varied from a maximum of 99% to < 0% in 10 sites where the nitrate–N load was greater than nitrate–N leached. The median nitrate–N retained was 50% (Fig. 1). The median N-retained for sites under MDC (25%) was less (*P* < 0.05; Mann–Whitney) than the median value for all other sites (52%). Regression analysis of nitrate–N retention indicated a significant (*P* = 0.015) but poorly fitting model (*R*^2^ = 0.12) with catchment area and mean altitude being the only significant predictors retained in the model.

### Lag times

**Figure 2:**
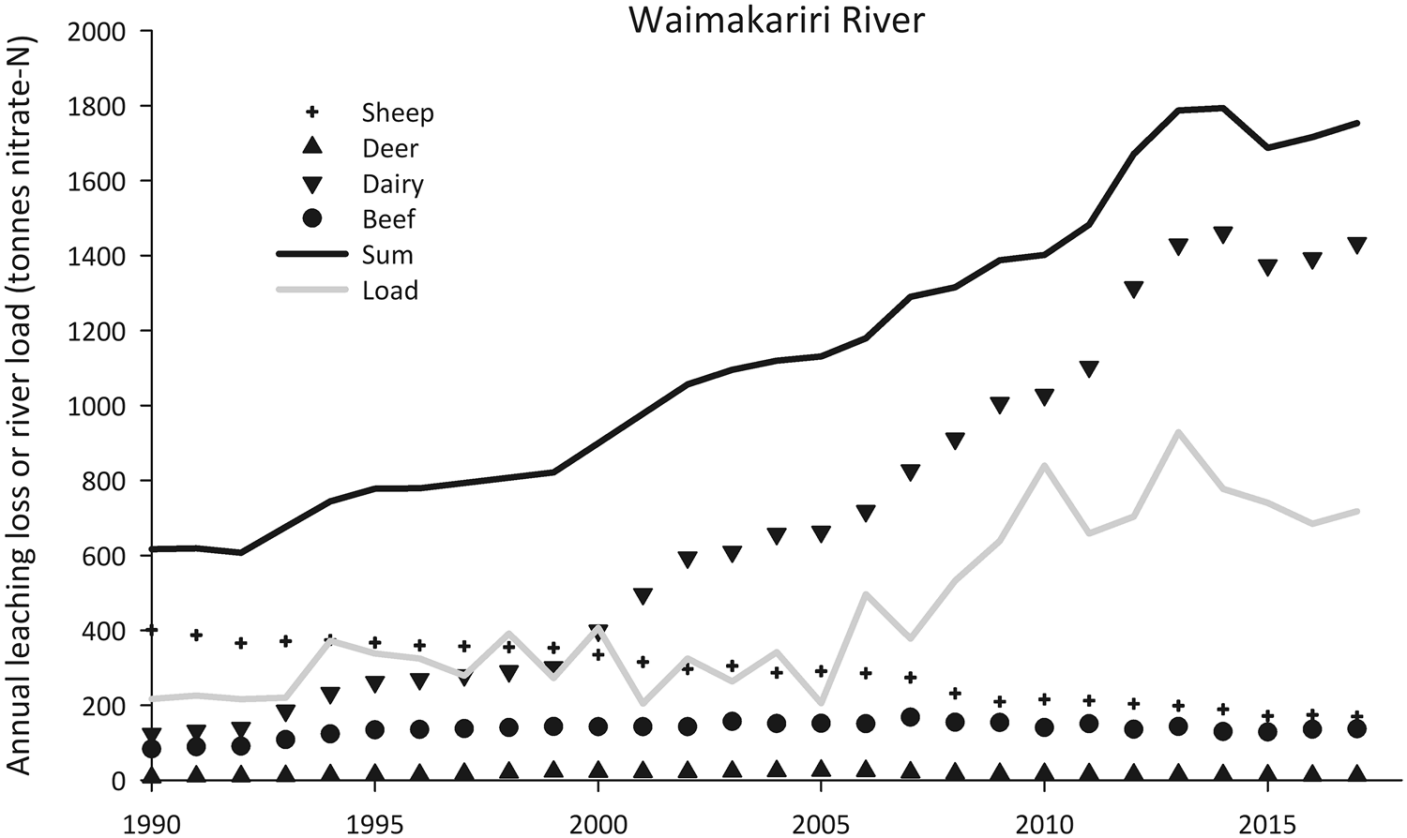
Annual leaching loss of nitrate–N (tonnes) over 1990–2018 for each livestock class, the sum of livestock classes and the load in the river for site CH4 on the Waimakariri River, Canterbury. Note that the increase in load occurs after the increase in nitrate–N leaching losses.

Over time, catchment nitrate–N loads have changed at a different rate relative to nitrate–N leaching losses. Nitrate–N loads have increased with intensification and expansion of, for example, dairy cattle numbers and coverage from ~ 3.2 M in 1990 to ~ 5.0 M in 2015^16^. An example is given in Fig. 2 for the Waimakariri River in Canterbury (site CH4) showing increasing leaching losses of nitrate–N from land grazed by dairy cattle, at the expense of losses from land grazed by sheep and beef cattle, and a commensurate increase in catchment nitrate–N load. Notably, the sum of nitrate–N leaching loss across the catchment increases before the catchment nitrate–N load increases.

The lag time between nitrate–N leaching losses and river loads was calculable for 49 of the Network catchments using at least one of two methods, a cross-correlation function and cumulative generalised additive models (cumulative GAMs). Lags could not be calculated for 28 catchments. However, of the 49 catchments, five were excluded from further analysis as they were deemed as ‘impacted’ by point sources or had highly modified flows caused by hydroelectricity generation, and 10 were excluded as they were under MDC and therefore unlikely to show any changes in anthropogenic inputs. Although these exclusions may have biased our estimates of median lag times, we have no evidence to say that their distribution of lag times would be any different to those included in further analysis. Indeed, a Mann–Whitney test of the coefficients of variation for nitrate–N leaching losses and loads showed no difference between median coefficient in those catchments where calculations could or could not establish lag times. This suggests that it was not the magnitude of change but when changes were occurring that was causing the detection (or not) of lag times. Supporting this, a Mann–Whitney test of the annual median slope for leaching losses over time showed those catchments with a lag time (1.445 tonnes yr^−1^) were significantly greater than those without lag times (0.0026 tonnes yr^−1^). The near zero increase in mean annual nitrate–N leaching losses is to be expected in catchments with no significant agricultural activity.

**Figure 3:**
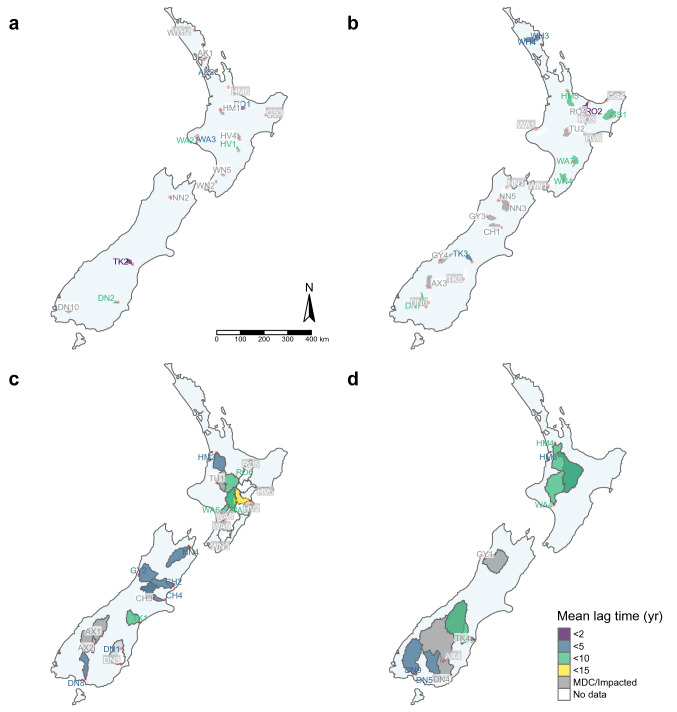
Map of New Zealand showing the location, catchment size and estimated lag time between nitrate–N leaching losses and nitrate–N load for catchments of: (**a**) < 450, (**b**) 451–1800, (**c**) 1801–5000, and (**d**) > 5000 km^2^. Red dots refer to the location of the sampling site. Note that some sites are subsumed within larger catchments of the same lag time class. Maps were created using R (ver. 4.0.3) packages 'sf'^86^ and 'tmap'^87^; New Zealand boundary data sourced from https://www.stats.govt.nz/ while catchment boundaries were sourced from the River Environment Classification database (https://niwa.co.nz/freshwater-and-estuaries/management-tools/river-environment-classification-0).

Significant lag times in nitrate–N loads were estimated for 34 catchments, 18 and 31 via the cross-correlation and cumulative GAM approaches, respectively; 12 catchments exhibited significant lag times using both approaches (Table 2, Fig. 3). Lag times using the cross-correlation approach were related to those using the cumulative GAM approach (Cross-correlation = 1.23 × Cumulative GAM − 3.2, R^2^ = 0.41, *P* < 0.019). The mean and median lag time across all 34 catchments were 5.1 and 4.5 years, respectively.

Regression analysis indicated that lag times between nitrate–N leaching losses and loads (of individual techniques or a mean where both returned a significant result) could be predicted (adjusted R^2^ = 0.47; Supplementary Table S2) with increasing mean catchment altitude (metres above sea level), stream order at the catchment outlet (Strahler), and deceasing mean slope (degrees), population density (people km^−2^) and geologic class (going from sedimentary to volcanic):

1 $$\begin{aligned} {\text{Lag }} & = \, - {9}.{1 } + \, 0.00{\text{85Mean Altitude }} + \, 0.{\text{592Stream order }}{-}{ 1}.0{\text{97Mean Slope }} \\ & \quad + \, 0.{\text{227Evapotranspiration }}{-} \, 0.0{\text{68Populaton density }}{-} \, 0.{\text{784Geology class}} \\ \end{aligned}$$

Amongst these parameters, the most variance was explained by mean altitude, mean slope, stream order, and evapotranspiration, with smaller contributions from geologic class, and population density. Including other parameters such as land use class, area, baseflow fraction, and rainfall resulted in small improvements in predictive power and an overfitted model.

### Hydrologic comparison of lag times

Mean transit time data for water (or hydrologic lag) were available for 14 catchments, eight of which had significant lag times estimated by either the cross-correlation or cumulative GAM approaches. Mean lag times in these eight catchments were all greater than or within the range of mean transit times for baseflow (Table 2), which is likely to be a combination of young, shallow groundwater and older interflow. On average, nitrate–N lag times in these nine catchments were 2.1 years longer than the mid-point of mean transit times for baseflow.

## Discussion

### N-retention

The range of N-retained in our catchments (− 160 to 99%) varied more than in other studies. For example, Dupas et al.^17^ noted retention percentages of 45–88% in 16 agricultural catchments in Brittany, France. A similar range (53 ± 24%) was noted for 160 catchments across wider France^18^, while a narrower range (65 to 90%) was noted in 16 catchments in the north-eastern US, with variation being attributed to their ‘wetness’^19^.

N-retention did not correlate to lag times and was only partly explained by an increase in area and mean altitude, presumably as leached nitrate–N was subjected to a longer and more tortuous route to the catchment outlet leading to more removal by processes like denitrification^20^. The absence of predictors like land use may reflect the fact that we included these as a static point in time, whereas they would be changing during the study. However, as different catchments would be changing at different times, no one metric to capture that change could be included in the model.

A negative N-retention indicates that more nitrate–N was leaving the catchment than was estimated to be leached. Negative N-retention percentages were largely confined to those in MDC. The negative N-retention from these catchments is likely caused by low agricultural activity and not including N inputs via atmospheric deposition or erosion, which in agricultural catchments would be obscured by N inputs from animals^21^. Parfitt et al.^22^ estimated losses via erosion and leaching of N in catchments under native forestry in New Zealand, i.e. MDC, to be about 3 kg N ha^−1^ yr^−1^, whereas atmospheric inputs in rainfall were 1.5 kg N ha^−1^ yr^−1^ except in catchments with high rainfall where inputs can be > 5 kg N ha^−1^ yr^−1^. On average, MDC catchments had a 350 m greater mean altitude and about a 50% greater rainfall than other catchments (see Supplementary data).

Amongst catchments that were impacted by agricultural activity, the range of N-retention (− 2 to 99%) was broader than found in other studies. As mentioned above, our predictor variables were largely unable to explain variation in N-retention. Other studies have focused on the interaction of catchment characteristics such as biogeochemistry^23^, and catchment management such as point sources, agricultural intensification^17,24^ and flow-paths^8,25^. In contrast to these studies, our catchments were more diverse in altitude, geology, and land use intensity (Table 1), and had little point source contributions^26^. For instance, the catchments studied by Dupas et al.^17^ had a rainfall range of 195–689 mm yr^−1^ and agricultural land use not less than 30%; ours ranged from 828–3696 mm and 0–99% (Table 1). Such diversity should generate greater predictive power, if spread linearly over the range of values. However, the large size of our catchments (Table 1) coupled with a large intra-catchment diversity in altitude, soil type, and climate implies that mean catchment variables may not be representative of the catchment processes that control N-retention, or that the interaction of these variables causes a lag in their effect. Indeed, long lag times of up to 34 years (mean of 5.5 years) caused by a diversity of flow paths was labelled as a cause of attenuation of nitrate–N loads in the large Grand River catchment (6800 km^2^) of southwestern Ontario^12^.

### Lag times

A handful of studies have used a data driven approach to establish lag times between nitrate–N inputs (or leaching losses) and nitrate–N loads in rivers. For instance, Van Meter and Basu^12^ found lag times varied from 12–34 years with longer lag times in the lower watershed corresponding to catchments that were dominated by groundwater flows. Similarly, transit time distributions (effectively lag times in the way they were assessed) of 2–14 years were calculated by Dupas et al.^17^ with longer lag times associated with catchments with granite and mixed-lithology than calculated for catchments with schist lithology. In a mixed forest/agricultural catchment, Ehrhardt et al.^24^ found lag times in nitrate–N ranging from 7–22 years depending on stream location and season as either shallow, proximal (shorter lag times; e.g. through riparian soils and tile drains) or deeper (longer lag times; e.g. aquifer-driven) flow-paths contributed the nitrate load.

Our data showed that a combination of mean altitude, mean slope, evapotranspiration, and stream order strongly influenced lag times, with weaker influence from geologic class and population density. An increase in lag time is expected as stream order increases^27^ because deeper flow-paths carrying older water will likely contribute more of the total stream flow. However, in catchments of comparative size but with greater mean altitude and slope, steeper slopes will promote deeper vertical infiltration resulting in a wider range of flow paths with different ages than in flatter areas, lengthening lag times^20,28^. Of the other variables, increasing lag times would be expected with decreasing population density which would act as a surrogate for increasing stream order and catchment size. Likewise, certain geologic classes can increase lag times, especially in catchments with porous bedrock like chalk^29^. Although limestone and chalk geology is rare in New Zealand, long lag times (70–110 years) have been noted for groundwater in catchments with porous volcanic geology^7^.

Nitrate–N lag times are likely longer than hydrologic lag (viz. transit) times, owing to the presence of biogeochemical lags that result in N being stored in the soil^30^. However, data is emerging to suggest that hydrologic lags dominate overall time lags in nitrate loads. This has been attributed to not only the mobile nature of nitrate–N but also to periods of sustained N inputs^24^ or of diminished biogeochemical N retention^4^—effectively surpassing the ability of soils and flow paths to remove added N. Dupas et al.^17^ found lag times that were equivalent to hydrologic times but noted that a biogeochemical lag was still likely which would lengthen the tail of nitrate–N delivery.

We made no attempt to differentiate between hydrologic and biogeochemical lags. However, owing to the presence of isotope and modelling data for nine of the catchments with nitrate–N lags we could infer that the nitrate–N lag was on average only 2.1 years longer than the hydrologic lag. However, data for hydrologic lag times were derived under baseflow conditions (except for CH4), meaning that younger water from surface runoff pathways would likely decrease the hydrologic lag. The baseflow index varied from 0.03 to 0.88 across all 77 catchments and from 0.17 to 0.50 in the eight catchments that had hydrologic lag times. Assuming water from surface runoff was at least half the age of groundwater (viz. baseflow)^31^, a mean calculated hydrologic lag time for these nine catchments would be 1.1, approximately 3.9 years younger than the mean nitrate–N lag.

### Limitations of lag time calculations

Our lag time calculations have limitations in both data and analytical approaches. Although nitrate–N leaching losses were calculated using software that has been developed and calibrated to nitrate–N losses in New Zealand^32^, the good spatial representativeness that was achieved at a farm scale during census years (varying from 3–6 years apart) reduced to trends in land use associated with a local government district level in other years. However, one benefit of calculating lag times in large catchments is that they often match district boundaries and land use change tends to be within rather than between catchments. Outside of pastoral land, our estimates of nitrate–N leaching losses may have also been hampered by the accuracy of export coefficients for other crops. However, their coverage and expansion over the last 30 years has been small (Supplementary Fig. S2).

Analytically, there are also limitations to the utility of the cross-correlation and cumulative GAM approaches to lag times. Considering cross-correlation, one limitation is that correlation does not confirm a causal link between catchment nitrate–N leaching losses and riverine nitrate–N loads^33^. Secondly, the cross-correlation approach depends on the *changes* in the two series to identify significant lags. Since our catchment N leaching losses were on an annual timestep, correlations were based on only 28 data points. Therefore, longer lags identified with cross-correlation will rely on fewer data points and require caution. A finer seasonal scale^24^ may have shown better results. Additionally, if gross nitrate–N leaching loss is relatively linear through time, then identifying the correlation between changes in the two series will be difficult for the catchment. Thirdly, this approach overlooks the cumulative nitrate–N leaching loss; riverine nitrate–N load is likely influenced by all the losses up to the characteristic lag time, not just nitrate–N losses at that lag.

While the cumulative GAM approach connects nitrate–N leaching losses to riverine loads in an adaptive statistical model, it too has limitations in estimating lag times. First, like the cross-correlation approach there are limited data, i.e. up to 28 annual values for each catchment. Second, our models only approximate the relationship between nitrate–N leaching losses and river loads. Our model^4^ attempts to increase realism by including lagged surplus precipitation as a predictor. Ideally, leaching losses would take the form of an *interaction* term between subsurface hydrologic flow and nitrate–N leached from the root zone and soil profile, thereby accounting for biogeochemical processes regulating nitrate–N availability but also the timing of subsurface flows. Rather, our data only permitted us to include the separate effects of cumulative excess precipitation in^4^. Third, in some cases, it was not possible to identify the smooth term *f*_4_ in^3,4^ due to limited observations or circumstances leading to poor identifiability (e.g., weak but consistent linear trends in leaching losses). Finally, our nitrate–N leaching losses consider denitrification in the topsoil but ignore the likely removal of nitrate–N in other pathways before leaving the catchment^34^. However, since we use cumulative catchment nitrate–N leaching losses to model the variability in riverine loads, we do not require a nitrate–N mass balance. Hence, we assume that variation in nitrate–N leaching losses is predictive of changes in river loads and are most predictive at the characteristic lag time for the catchment.

### Impact on local policy development

Policy to protect water quality requires landowners and managers to put in place actions to mitigate leaching losses of nitrate–N to decrease the load of nitrate–N in receiving waterbodies. In New Zealand, a recent analysis found that 43% of agricultural land was in catchments where the current load exceeded the maximum allowed^35^. Other work^36^ estimated that, had farming mitigation practices over 1995–2015 not been adopted, 45% more N (largely as nitrate–N) would have been lost. Despite these efforts, the expansion of intensive land uses has increased N loads by 25% nationally^36^. Where it was assumed all actions to mitigate N losses were adopted, additional modelling showed that future N loads could decrease by about a third^37^, reducing the area still exceeding the maximum allowed to about 5%^38^. However, this modelling assumes that actions were adopted over a period of 20 years, commensurate with the mean rate of adopting agricultural practice in Australasia of about 17 years^39^. Government policy aims to show improvements in water quality metrics within five years and to make waterways healthy within a generation^4^. Although policy can enforce action to occur quickly, it is still likely that their implementation and effectiveness will take time to reach their full potential. Our work would suggest that, with a median lag time in nitrate loads of 4.5 years, targeted improvement would not be possible in nearly half of our catchments, which are representative of agricultural land use in New Zealand. It is likely that small catchments or sub-catchments of those with longer lag times would respond quicker. Improving the estimation of such changes could be aided with adjustments of the current monitoring network^40,41^.

## Materials and methods

### Nitrate–N leaching estimates

Annual estimates of nitrate–N leaching loss were generated nationally using the method of Dymond et al.^42^. Briefly, this method combines farm level data for livestock class (beef cattle, dairy cattle, sheep and deer) and livestock numbers, collected approximately during agricultural censuses (1994, 2002, 2007, 2012, and 2017)^43^, with modelled estimates of nitrate–N loss from the root zone and soil profile (via the model OVERSEER^44^) for those livestock numbers and classes across 100 unique soil by climate combinations identified at level II of the Land Environments of New Zealand spatial database^45^. Annual counts of livestock types in-between census years were taken from district level data (n = 53). These data were allocated to pastoral land uses according to the Land Cover Database (1997/98, 2001/02, 2008/09, 2012/13, 2018/19)^46^ and then to properties using AgriBase^47^. It was assumed that changes between census years were proportional to livestock classes and numbers as indicated across farm types within a district. For example, if dairy cattle numbers increased by 50% between two censuses, the increase was apportioned equally to all dairy farms identified by the Land Cover Database and AgriBase within that district.

Arable and horticultural land uses occupy small areas of New Zealand (c. 0.5Mha *cf* 11.4 M ha for cattle, sheep and deer^48^). Few data exist or can be modelled successfully for the wide range of arable and horticultural crops and crop-rotations used year-to-year. Hence, we used a constant nitrate–N loss estimate of 30 kg N ha^−1^ yr^−1^ for arable and horticultural land based on the median of field studies^49^. Inputs from native forestry were set at 1.5 kg N ha^−1^ yr^−1^^22^.

### Catchment concentration and discharge data

We calculated annual nitrate–N loads for 77 sites from 1989–2020 from the National River Water Quality Network run by the National Institute of Water and Atmospheric Research (NIWA) and Regional Councils in New Zealand. The Network is located on 48 of New Zealand’s rivers covering a range of flow regimes, catchment characteristics, and land use. Some rivers contain more than one site. We refer to a site as a river with its own catchment. No significant point sources are included on the network. The approach used a GAM to predict daily loads from monthly nitrate–N concentration measurements and daily mean discharge, accounting for the time of year and flow regime. Daily loads were summed to annual loads.

All monthly nitrate–N concentration data were sourced from NIWA. Data were also secured from NIWA for daily mean discharge (calculated from 15 min observations) at each of these rivers from 1989–2010 and for 33 rivers from 2010–2020. Discharge for the remaining 44 rivers from 2010–2020 were sourced from a combination of Regional Councils and hydroelectric power producers. A description of the rivers, methods used, and quality of the data are available elsewhere^6,50,51^.

Gaps in the stream discharge records were < 1% of all data with maximum lengths of consecutive missing data < 40 days for 63 sites but 51 to 432 days for 9 rivers. To infill gaps we imputed values using the ‘GR4J’ hydrological model^52^ via the ‘airGR’ package in R^53^. This rainfall-runoff model used daily gridded precipitation and potential evapotranspiration (Penman method), sourced from the National Virtual Climate Station network^54^ that were subsequently averaged across catchments with inverse distance weighted interpolation.

Discharge flow components (‘baseflow’ and ‘quick flow’) were identified at each river with the hydrograph separation technique detailed by Pelletier and Andréassian^55^. We note that some catchments are subject to dam regulation and/or glacial melt (e.g., in the Otago region), meaning that ‘baseflow’ for these catchments will be an arbitrary, slow component of mean daily discharge (*Q*); consequently we de-emphasize baseflow effects for such catchments. For the entire record, baseflow fraction was calculated as daily baseflow divided by daily *Q*.

### Estimating catchment nitrate loads

We calculated nitrate–N loads (kg N d^−1^) for dates with grab samples as nitrate–N concentration times daily *Q*. Generally, concentrations were above detection limits (1 µg N L^−1^); however, three rivers had 20–46% of observations at or below detection limits (up to ~ 7% of observations for 31 other rivers). While censored values can bias statistical models at low ranges of concentration^56^, we consider this a minor problem for our objective of determining annual nitrate–N loads and long-term trends in stream nitrate–N load regimes, where the majority of load is typically delivered during storm events with concentrations orders of magnitude greater than detection limits (see Supplementary Fig. S3). Hence, we used half the detection limit for censored observations and accept the minor amount of bias this has on our load models.

To model stream nitrate–N loads, we used concentration data to fit GAMs^57^ for each river. Our approach is closely related to the framework of Hirsch et al.^58^ but allows more flexible model building and testing. We explored several variants of the model, but found the following to be most generally applicable:

2 $$\begin{aligned} & {\text{g}}\left( {\upmu } \right) = {\upalpha } + f_{1} \left( {log\left( Q \right),Base\; flow\; fraction} \right) + f_{2} \left( {DOY} \right) + f_{3} \left( t \right) \\ & y \sim {\Gamma }\left( {{\upmu },{\uplambda }} \right) \\ \end{aligned}$$

where µ is the conditional expected nitrate–N load, the link function used (*g*) is the log-link, α is an intercept term, *f*_1_–*f*_3_ are smooth functions (see below) of the predictors, and the observed data (*y*) are modelled as gamma-distributed with mean µ and scale λ. Notably, the gamma distribution with the log-link: (1) accounts for the heteroskedasticity common in many water quality data, since λ can vary linearly with the magnitude of the load (µ), and (2) easily allows for predictions of nitrate–N load on the original response scale without the need for bias-correction when back-transforming predictions on the log scale^59^. Alternative distributions (e.g., log-normal) yielded poorer fits.

We chose *f*_3_ to be a thin-plate regression spline function of time (*t*) to account for non-linear and variable trends in nitrate–N load over the ~ 30-year period. We modelled seasonality with a cyclic-cubic spline (*f*_2_) of day-of-year (DOY; 1 to 365/366). Finally, since nitrate–N concentrations typically vary with flow (*Q*) but also depend on whether the flow is derived from a recent storm event (low baseflow fraction) or a longer-term recession (higher baseflow fraction), we modelled this interaction between baseflow fraction and log-*Q* with a bivariate tensor product smooth (*f*_1_). This term comprises a thin-plate regression spline for log-*Q* and a cubic-regression spline for baseflow fraction plus the interaction between the two smooth functions. For choice of smoother used, we generally opted for the default thin-plate regression spline or, if the predictor was cyclical, the cyclic-cubic spline but opted for a cubic-regression spline for some terms (e.g., for baseflow fraction in the tensor product smooth) to reduce computational cost; while thin-plate regression splines are more robust^57^, the choice of a cubic-regression spline for well-constrained variables such as baseflow fraction did not significantly influence the fit.

Through prior experience, literature review^56^, and exploratory analyses, we deemed this model structure sufficient and robust in capturing the dominant features of nitrate–N load in these streams. More complicated models (e.g., where the concentration-discharge relationship captured in *f*_1_ could also itself vary with time^58^) are possible, data permitting, but, in general, few cases warranted the more complex structure, delivering only marginal improvements in predictive performance (below) over the more parsimonious model (this is explored and output in the Figshare repository: see Data availability section). This simpler GAM fit had significant effects (using the approximate *p*-value for GAM smooth components^60^) for all terms for 70 of the 77 rivers; the remaining rivers had marginally low *p*-values (up to *p* = 0.15) for either the time trend or seasonal component but, for simplicity, these minimally influential terms were kept in the model for these rivers.

The GAMs were fitted via restricted maximum likelihood (REML) with the ‘mgcv’ package within R^61^. We assessed each fit with various residual diagnostic plots. When trialling alternative model fits, we compared models via the Akaike information criterion (AIC), residual diagnostics, and posterior simulation behaviour (e.g., to check for over-fitting).

Annual nitrate–N loads were calculated by predicting daily nitrate–N load across the record and summing for each year. The uncertainty in these annual loads was estimated by drawing 1000 random sets of parameters from the posterior distribution of the parameters in the original GAM, re-estimating the annual load with these simulated parameters, and summarizing these simulations with a 95% credible interval^57,61^.

### Calculating lag-times between nitrate leaching and catchment loads

To estimate the potential lag time between nitrate–N leaching losses and riverine nitrate–N loads, we conducted three different analyses: (1) cross-correlation, (2) cumulative GAMs, and (3) assessed mean hydrologic transit times from modelling and isotope data. Each has its strengths and weaknesses (see “Discussion”), but we synthesize all three approaches when discussing lags in nitrate–N delivery in our catchments.

#### Cross correlation analysis

Recent work^12,62^ assessed time lags in catchment N loads by pairing time series of catchment N inputs and N outputs (usually on an annual time step) and calculating the cross-correlation function for each lag time of interest (in years). This cross-correlation is the Pearson correlation coefficient between annual catchment nitrate–N load, *y*_*t*_, and the preceding (lagged) annual catchment nitrate–N leaching losses, *x*_t-k_, where *k* is lag in years from 1 to some maximum, *k*_max_. Hence, if *changes* in the riverine nitrate–N load regime lags *changes* in catchment nitrate–N leaching losses by *k* = 5 years, we expect the cross-correlation to reach a maximum near *k* = 5. Here, our *x*_t_ series is the annual nitrate–N leaching losses and our *y*_t_ is the annual nitrate–N load normalized for effects of all variables *except* time, i.e. analogous to the ‘flow-normalized’ loads in Hirsch et al.^58^, which corrects the nitrate–N loads for the variability in those exogenous predictor variables.

It is necessary to ‘pre-whiten’ both time-series before calculating the cross-correlation^63^ (though some authors argue against this^12^). Pre-whitening ensures that potential autocorrelation and non-stationarity within the two series do not falsely produce significant cross-correlations. To pre-whiten, we first-order differenced both *x*_t_ and *y*_t_ and then fitted an auto-regressive integrated moving average (ARIMA) model to the differenced *x*_t_. We used the automatic procedure for fitting a suitable ARIMA developed by Hyndman and Khandakar in the ‘forecast’ package^64^. The resultant ARIMA model was then used to filter both differenced *x*_t_ and *y*_t_ prior to calculating the cross-correlation^63^. We calculated cross-correlations up to a *k*_max_ of 20 years.

#### Cumulative GAMs

Recognizing the limitations of the cross-correlation approach, we also modelled riverine nitrate–N loads using lagged, cumulative nitrate–N leaching losses as predictors by modifying the GAM in Eq. (2) to:

3 $${\text{g}}\left( {\upmu } \right) = {\upalpha } + f_{1} \left( {log\left( Q \right),Baseflow\; fraction} \right) + f_{2} \left( {DOY} \right) + f_{4} \left( {L_{k} } \right)$$

We substitute out the time trend component (*f*_3_) for a smooth function of cumulative nitrate–N leaching losses for some lag of *k* years (*L*_k_). Our hypothesis with this change is that, with other dominant features of nitrate–N loads controlled for via *Q*, baseflow fraction, and DOY, the main driver behind changes in the nitrate–N loads is the change in cumulative nitrate–N leaching losses. In essence, the best lagged cumulative nitrate–N leaching losses predictor, *L*_*k*_, will best account for the smooth time trend in the original model fit.

We also modified this GAM to account for climatic variability where, even if there is considerable nitrate–N leaching lost from the root zone and soil profile but relatively little surplus rainfall, the potential for leached nitrate–N reaching the stream is low:

4 $$g\left( {\upmu } \right) = \alpha + f_{1} \left( {log\left( Q \right),Baseflow\; fraction} \right) + f_{2} \left( {DOY} \right) + f_{4} \left( {L_{k} } \right) + f_{5} \left( {S_{k} } \right)$$

where *S*_*k*_ is the cumulative sum of daily precipitation minus potential evapotranspiration (mm) for the preceding period of *k* years. Hence *S*_*k*_ provides a reasonable proxy for hydrologic conditions in the catchment for the lag *k* considered.

Both *f*_4_ and *f*_5_ were incorporated as smooth functions in the GAMs. While we used a thin-plate regression spline for *f*_5_, we used a shape-constrained spline for *f*_4_ (see Pya and Wood^65^) to avoid the possibility of negative loads. We found that unconstrained fits for the *L*_*k*_ smooth could sometimes be unrealistic (e.g., decreasing riverine nitrate–N load despite greater *L*_*k*_)—this was usually due to lack of observations to inform the fit. Based off previous studies of catchment nitrate–N dynamics^66–68^, we would strongly expect riverine nitrate–N loads to only increase or level off with increasing *L*_*k*_. We therefore enforced this constraint by modelling *f*_4_ as a monotonic-increasing function with the ‘scam’ package in R^65^.

Using the cumulative GAMs Eqs.( 3) and (4), we test *k* lags for the *L*_*k*_ predictor of 1 to 10 years. At a given lag, we fit Eqs. (3) and (4) where *L*_*k*_ and *S*_*k*_ are their cumulative sums for that lag. As a comparison, we also re-fit our original model Eq. (2) since the underlying data available becomes shorter as the considered lag becomes longer. We extract the effective degrees of freedom and approximate *p*-value for the smooth term for *L*_*k*_ (*f*_4_) in Eqs. (3) and (4) as well as all models’ AIC. We then make relative comparisons between these models at each lag by subtracting the AIC of Eqs. (3) and (4) from the AIC of Eq. (2): a ‘rule of thumb’ here is that a model outcompetes another candidate model when its AIC is more than 2 units lower^69^. We note that lags with well-performing models of either Eqs. (3) or (4) tended to have effective degrees of freedom of *f*_4_ closer to a value of 1 (nearly linear): these model fits favoured simple functions of *L*_*k*_ for predicting riverine nitrate–N loads.

For each river, we present the mean of the two lags with the lowest AIC (> 2 units from the fit of Eq.( 2)) and *P* value < 0.1 as the output of the cumulative GAM.

#### Mean transit time from isotope data

Estimates of hydrologic mean transit time for surface waters were sourced from the literature. These estimates place a minimum bound for lag time for water and hence nitrate–N for nine of the catchments. They were derived by a combination of different model types (e.g., binary mixing, dispersion, and exponential-piston flow) and isotope (e.g., ^3^H, ^18^O) data from water samples taken at baseflow for eight catchments and a combination of baseflow and stormflow for one catchment (CH4).

### Presentation of lags

We present mean lag times calculated from significant fits (*P* < 0.1) of the cross-correlation and cumulative GAM functions. We did not calculate lag times for those catchments where intensive agriculture was < 10% of the catchment’s area. These catchments are under MDC (viz. reference conditions) and are unlikely to exhibit significant nitrate–N leaching^70^. Land use in these catchments has not changed > 0.25% over the period of monitoring (Table 2 and Supplementary Fig. S2). We also filtered out rivers that were either impacted by hydroelectric schemes or had significant land works or forestry harvesting that caused uncharacteristically high sediment load^50^ (Table 2).

To model mean lag times as a function of catchment attributes, we obtained data for 13 variables that described climate, hydrology, and land use at a catchment level (Table 1). These data were used in a best subsets regression to output an ‘optimal’ candidate based on maximising the adjusted R^2^, minimising the AIC, and exhibited a Mallows Cp value that closely matched the number of predictor variables to avoid overfitting.

In addition to calculating and predicting lag times we calculated the cumulative nitrate–N retained in a catchment over the period of record as the difference between annual nitrate–N leached relative to annual nitrate–N load in the river as:

5 $${\text{Retention }} = 100\% \times \left( {1 - \frac{{\mathop \sum \nolimits_{i = 1989}^{2018} Load_{i} }}{{\mathop \sum \nolimits_{i = 1989}^{2018} Leaching_{i} }}} \right)$$

Nitrate–N retained is inclusive of loss processes such as denitrification in aquifers^71^ or uptake by stream vegetation and benthic sediments^72^ but excludes input processes such as atmospheric deposition. A similar approach has been used at the catchment level in other regions and globally^17,73^. We used the same input variables and analysis used to predict mean lag times to predict nitrate–N retained.

## Supplementary Information

Supplementary Information.

## Data Availability

R code for nitrate–N loads and lag-times and the datasets generated during the current study are available in the *Figshare* repository: 10.6084/m9.figshare.14576217.v1. Raw data for nitrate–N leaching losses and catchment nitrate–N concentrations and discharge are available from the New Zealand Ministry for the Environment and the National Institute for Water and Atmospheric Research, respectively.

## References

[1] Wang L, Butcher AS, Stuart ME, Gooddy DC, Bloomfield JP (2013). The nitrate time bomb: a numerical way to investigate nitrate storage and lag time in the unsaturated zone. Environ. Geochem. Health.

[2] Bieroza M (2019). Hydrologic extremes and legacy sources can override efforts to mitigate nutrient and sediment losses at the catchment scale. J. Environ. Qual..

[3] Davey AJH (2020). Water quality benefits from an advice-led approach to reducing water pollution from agriculture in England. Agric. Ecosyst. Environ..

[4] Ministry for the Environment. Essential Freshwater: Overview factsheet. 7 (Ministry for the Environment, Wellington, New Zealand, 2020).

[5] Carvalho L (2019). Protecting and restoring Europe's waters: An analysis of the future development needs of the Water Framework Directive. Sci. Total Environ..

[6] Snelder TH, McDowell RW, Fraser CE (2017). Estimation of catchment nutrient loads in New Zealand using monthly water quality monitoring data. JAWRA J. Am. Water Res. Assoc..

[7] Morgenstern U, Daughney CJ (2012). Groundwater age for identification of baseline groundwater quality and impacts of land-use intensification: The National Groundwater Monitoring Programme of New Zealand. J. Hydrol..

[8] Woodward SJR, Stenger R (2018). Bayesian chemistry-assisted hydrograph separation (BACH) and nutrient load partitioning from monthly stream phosphorus and nitrogen concentrations. Stoch. Env. Res. Risk Assess..

[9] Burow KR, Nolan BT, Rupert MG, Dubrovsky NM (2010). Nitrate in groundwater of the United States, 1991–2003. Environ. Sci. Technol..

[10] Woodward SJR, Stenger R, Hill RB (2016). Flow stratification of river water quality data to elucidate nutrient transfer pathways in mesoscale catchments. Trans. ASABE.

[11] Clague JC, Stenger R, Morgenstern U (2019). The influence of unsaturated zone drainage status on denitrification and the redox succession in shallow groundwater. Sci. Total Environ..

[12] Van Meter KJ, Basu NB (2017). Time lags in watershed-scale nutrient transport: an exploration of dominant controls. Environ. Res. Lett..

[13] Ali G, Oswald C, Spence C, Wellen C (2018). The T-TEL method for assessing water, sediment, and chemical connectivity. Water Resour. Res..

[14] Davies-Colley RJ (2011). Twenty years of New Zealand’s national rivers water quality network: Benefits of careful design and consistent operation1. JAWRA J. Am. Water Res. Assoc..

[15] Statistics New Zealand. *Agriculture, horticulture, and forestry*, <http://archive.stats.govt.nz/browse_for_stats/industry_sectors/agriculture-horticulture-forestry.aspx> (2018).

[16] DairyNZ. 2015/16 New Zealand Dairy Statistics. (DairyNZ, Hamilton, New Zealand, 2016).

[17] Dupas R, Ehrhardt S, Musolff A, Fovet O, Durand P (2020). Long-term nitrogen retention and transit time distribution in agricultural catchments in western France. Environ. Res. Lett..

[18] Dupas R (2015). Assessing the impact of agricultural pressures on N and P loads and eutrophication risk. Ecol. Indicators.

[19] Howarth, R. W. *et al.* in *Nitrogen Cycling in the Americas: Natural and Anthropogenic Influences and Controls* (eds Luiz A. Martinelli & Robert W. Howarth) 163–186 (Springer Netherlands, 2006).

[20] Vero SE (2017). A framework for determining unsaturated zone water quality time lags at catchment scale. Agric. Ecosyst. Environ..

[21] Heggie K, Savage C (2009). Nitrogen yields from New Zealand coastal catchments to receiving estuaries. N. Z. J. Mar. Freshwat. Res..

[22] Parfitt RL, Schipper LA, Baisden WT, Elliott AH (2006). Nitrogen inputs and outputs for New Zealand in 2001 at national and regional scales. Biogeochemistry.

[23] Bouwman, A. F. *et al.* Global trends and uncertainties in terrestrial denitrification and N2O emissions. *Phil. Trans. R. Soc. B Biol. Sci.* **368** (2013). 10.1098/rstb.2013.0112 PMC3682736 23713114

[24] Ehrhardt S, Kumar R, Fleckenstein JH, Attinger S, Musolff A (2019). Trajectories of nitrate input and output in three nested catchments along a land use gradient. Hydrol. Earth Syst. Sci..

[25] Yang J, Heidbüchel I, Musolff A, Reinstorf F, Fleckenstein JH (2018). Exploring the dynamics of transit times and subsurface mixing in a small agricultural catchment. Water Resour. Res..

[26] Snelder TH, Larned ST, McDowell RW (2018). Anthropogenic increases of catchment nitrogen and phosphorus loads in New Zealand. N. Z. J. Mar. Freshwat. Res..

[27] Van Meter KJ, Basu NB, Van Cappellen P (2017). Two centuries of nitrogen dynamics: Legacy sources and sinks in the Mississippi and Susquehanna River Basins. Global Biogeochem. Cycles.

[28] Jasechko S, Kirchner JW, Welker JM, McDonnell JJ (2016). Substantial proportion of global streamflow less than three months old. Nat. Geosci..

[29] Ascott MJ, Wang L, Stuart ME, Ward RS, Hart A (2016). Quantification of nitrate storage in the vadose (unsaturated) zone: A missing component of terrestrial N budgets. Hydrol. Process..

[30] Basu, N. B. *et al.* Nutrient loads exported from managed catchments reveal emergent biogeochemical stationarity. *Geophys. Res. Lett.* **37** (2010).

[31] Stewart MK (2012). A 40-year record of carbon-14 and tritium in the Christchurch groundwater system, New Zealand: Dating of young samples with carbon-14. J. Hydrol..

[32] Shepherd, M., Wheeler, D., Freeman, M. & Selbie, D. Rationale for OVERSEER® Nutrient Budgets evaluation and recalibration. Report No. RE500/2015/034, (2015).

[33] Chatfield C (2003). The Analysis of Time Series: An Introduction.

[34] Schlesinger WH, Bernhardt ES (2020). Biogeochemistry.

[35] Snelder TH, Whitehead AL, Fraser C, Larned ST, Schallenberg M (2020). Nitrogen loads to New Zealand aquatic receiving environments: comparison with regulatory criteria. N. Z. J. Mar. Freshwat. Res..

[36] Monaghan R (2021). Quantifying contaminant losses to water from pastoral landuses in New Zealand II. The effects of some farm mitigation actions over the past two decades. N. Z. J. Agric. Res..

[37] McDowell RW (2021). Quantifying contaminant losses to water from pastoral land uses in New Zealand III. What could be achieved by 2035?. N. Z. J. Agric. Res..

[38] McDowell RW, Pletnyakov P, Lim A, Salmon G (2020). Implications of water quality policy on land use: a case study of the approach in New Zealand. Mar. Freshwater Sci..

[39] Kuehne G (2017). Predicting farmer uptake of new agricultural practices: A tool for research, extension and policy. Agric. Syst..

[40] Parliamentary Commissioner for the Environment. Focusing Aotearoa New Zealand’s environmental reporting system. 106 (Parliamentary Commissioner for the Environment, Wellington, New Zealand, 2019).

[41] Wellen C, Van Cappellen P, Gospodyn L, Thomas JL, Mohamed MN (2020). An analysis of the sample size requirements for acceptable statistical power in water quality monitoring for improvement detection. Ecol. Indicators.

[42] Dymond JR, Ausseil AG, Parfitt RL, Herzig A, McDowell RW (2013). Nitrate and phosphorus leaching in New Zealand: A national perspective. N. Z. J. Agric. Res..

[43] Statistics New Zealand. *Livestock numbers*, https://statisticsnz.shinyapps.io/livestock_numbers/ (2019).

[44] Wheeler, D. Overseer technical manual. Technical manual for the description of the Overseer nutrient budgets engine. Characteristics of animals. . (Overseer Ltd, Wellington, New Zealand, 2016).

[45] Leathwick, J. R. *et al. Land Environments of New Zealand: A Technical Guide*. 244 (Ministry for the Environment, 2002).

[46] Landcare Research. *NZ Land Cover Database*, http://www.lcdb.scinfo.org.nz/home (2017).

[47] Sanson, R. The Agribase^TM^ farm location database. *Proc. N. Z. Soc. Animal Prod.* **65** (2005).

[48] Ministry for the Environment & Statistics New Zealand. *Agricultural and horticultural land use*, http://infoshare.stats.govt.nz/browse_for_stats/environment/environmental-reporting-series/environmental-indicators/Home/Land/land-use.aspx (2020).

[49] Norris, M. *et al.* in *Science and policy: nutrient management challenge for the next generation* Vol. Occasional Report No. 30 (eds L. D. Currie & M.J. Hedley) 10 (Fertilizer and Lime Research Centre, Massey University, Palmerston North, New Zealand, 2017).

[50] Smith DG, McBride GB, Bryers GG, Wisse J, Mink DFJ (1996). Trends in New Zealand's national river water quality network. N. Z. J. Mar. Freshwat. Res..

[51] Julian JP, de Beurs KM, Owsley B, Davies-Colley RJ, Ausseil AGE (2017). River water quality changes in New Zealand over 26 years: Response to land use intensity. Hydrol. Earth Syst. Sci..

[52] Perrin C, Michel C, Andréassian V (2003). Improvement of a parsimonious model for streamflow simulation. J. Hydrol..

[53] Coron L, Thirel G, Delaigue O, Perrin C, Andréassian V (2017). The suite of lumped GR hydrological models in an R package. Environ. Model. Software.

[54] Tait A, Henderson R, Turner R, Zheng X (2006). Thin plate smoothing spline interpolation of daily rainfall for New Zealand using a climatological rainfall surface. Int. J. Climatol..

[55] Pelletier A, Andréassian V (2020). Hydrograph separation: an impartial parametrisation for an imperfect method. Hydrol. Earth Syst. Sci..

[56] Helsel, D. R., Hirsch, R. M., Ryberg, K. R., Archfield, S. A. & Gilroy, E. J. Statistical methods in water resources. Report No. 4-A3, 484 (Reston, VA, 2020).

[57] Wood, S. N. *Generalized Additive Models: An Introduction with R*. 2nd edn, 496 (CRC Press, 2017).

[58] Hirsch RM, Moyer DL, Archfield SA (2010). Weighted regressions on time, discharge, and season (WRTDS), with an application to Chesapeake Bay river inputs1. JAWRA J. Am. Water Res. Assoc..

[59] Hirsch RM, Slack JR, Smith RA (1982). Techniques of trend analysis for monthly water quality data. Water Resour. Res..

[60] Wood SN (2012). On p-values for smooth components of an extended generalized additive model. Biometrika.

[61] Wood SN (2011). Fast stable restricted maximum likelihood and marginal likelihood estimation of semiparametric generalized linear models. J. Roy. Stat. Soc. Ser. B. (Stat. Method.).

[62] Chen D, Huang H, Hu M, Dahlgren RA (2014). Influence of lag effect, soil release, and climate change on watershed anthropogenic nitrogen inputs and riverine export dynamics. Environ. Sci. Technol..

[63] Cryer JD, Chan K-S (2008). Time Series Analysis with Applications in R.

[64] Hyndman RJ, Khandakar Y (2008). Automatic time series forecasting: The forecast package for R. J. Stat. Softw..

[65] Pya N, Wood SN (2015). Shape constrained additive models. Stat. Comput..

[66] Van Meter KJ, Basu NB (2015). Catchment legacies and time lags: A parsimonious watershed model to predict the effects of legacy storage on nitrogen export. PLoS ONE.

[67] Sebilo M, Mayer B, Nicolardot B, Pinay G, Mariotti A (2013). Long-term fate of nitrate fertilizer in agricultural soils. Proc. Natl. Acad. Sci..

[68] Sudduth EB, Perakis SS, Bernhardt ES (2013). Nitrate in watersheds: Straight from soils to streams?. J. Geophys. Res. Biogeosci..

[69] Burnham KP, Anderson DR (2004). Multimodel inference: Understanding AIC and BIC in model selection. Soc. Methods Res..

[70] McDowell RW, Snelder TH, Cox N, Booker DJ, Wilcock RJ (2013). Establishment of reference or baseline conditions of chemical indicators in New Zealand streams and rivers relative to present conditions. Mar. Freshwat. Res..

[71] Singh R, Horne DJ (2020). Water-quality issues facing dairy farming: potential natural and built attenuation of nitrate losses in sensitive agricultural catchments. Animal Prod. Sci..

[72] Abell JM, Hamilton DP, Rutherford JC (2013). Quantifying temporal and spatial variations in sediment, nitrogen and phosphorus transport in stream inflows to a large eutrophic lake. Environ. Sci. Process Impacts.

[73] Ascott MJ (2017). Global patterns of nitrate storage in the vadose zone. Nat. Commun..

[74] Sayre, R. *et al.* A New Map of Global Ecological Land Units - An Ecophysiographic Stratification Approach. 46 (Association of American Geographers, Washington D.C., 2014).

[75] United States Department of the Interior - United States Geological Survey. HydroSHEDS. (U.S. Dept. of the Interior, U. S. Geological Survey, Washinton D.C., 2008).

[76] Center for International Earth Science Information Network - CIESIN - Columbia University & Centro Internacional de Agricultura Tropical - CIAT. Gridded Population of the World, Version 3 (GPWv3): Population Density Grid. (2005).

[77] Fekete, B. M., Vörösmarty, C. J. & Grabs, W. *UNH/GRDC Composite Runoff Fields v1.0*, http://www.compositerunoff.sr.unh.edu/ (2018).

[78] McDowell RW, Noble A, Pletnyakov P, Haggard BE, Mosley LM (2020). Global mapping of freshwater nutrient enrichment and periphyton growth potential. Sci. Rep..

[79] European Space Agency. *European Space Agency GlobCover Portal - GlobCover 2009*, http://due.esrin.esa.int/page_globcover.php (2010).

[80] Ministry for the Environment. *Freshwater classification system: River environment classification* https://www.mfe.govt.nz/environmental-reporting/about-environmental-reporting/classification-systems/fresh-water.html (2013).

[81] Willmott, C. J. & Matsuura, K. *Terrestrial Air Temperature and Precipitation: Monthly and Annual Time Series (1950 - 1999)*, http://climate.geog.udel.edu/~climate/html_pages/download.html (2001).

[82] Daughney, C. *et al.* Hydrochemistry of the Southland Region. 203 (GNS Science, Lower Hutt, New Zealand, 2015).

[83] Wilson, S., Chanut, P., Rissmann, C. & Ledgard, G. Estimating Time Lags for Nitrate Response in Shallow Southland Groundwater. 51 (Environment Southland, Invercargill, New Zealand, 2014).

[84] Morgenstern, U. *et al.* Heretaunga Plains Aquifers: Groundwater Dynamics, source and Hydrological Processes as Inferred from Age, Chemisty, and Stable Isotope Tracer Data. 82 (GNS Science, Lower Hutt, New Zealand, 2018).

[85] Morgenstern, U. *et al.* Ohau and Waikawa catchment of the Horowhenua Groundwater Management Zone - Groundwater dynamics, source, and hydrological processes as inferred from the groundwater tracer data. 52 (GNS Science, Lower Hutt, New Zealand, 2019).

[86] Pebesma E (2018). Simple features for R: Standardized support for spatial vector data. R J..

[87] Tennekes M (2018). tmap: Thematic maps in R. J. Stat. Softw..

