# Supplementary material for: The implications of lag times between nitrate leaching losses and riverine loads for water quality policy
Source: Sci Rep. 2021 Aug 12;11:16450. doi: 10.1038/s41598-021-95302-1 (PMC8360963; doi:10.1038/s41598-021-95302-1)
Supplement: Supplementary file 1 — Supplementary Information. [file 41598_2021_95302_MOESM1_ESM.docx]

**Supplementary Information:**

**The implications of lag times between nitrate leaching losses and riverine loads for water quality policy**

R. W. McDowell^1,2,*^, Z. P. Simpson^1^, A.G. Ausseil^3^, Z. Etheridge^4^, R. Law^5^

^1^Department of Soil and Physical Sciences, Lincoln University, Lincoln, New Zealand.

^2^AgResearch, Lincoln Science Centre, Lincoln, New Zealand

^3^Manaaki Whenua Landcare Research, 17 Whitmore St, Wellington, New Zealand

^4^Kōmanawa Solutions Ltd, Christchurch, New Zealand

^5^Manaaki Whenua Landcare Research, Private Bag 11052 Manawatu Mail Centre, Palmerston North, New Zealand

**Supplementary information**

**Table S1**. Catchment size, nitrate-N leached (1990-2018) by land use, the nitrate-N load (1990-2018; ± 95% confidence intervals, CI) of each river and the nitrate-N retained in the catchment.

| Site | Catchment size (Km^2^) | ---------------------------- Nitrate-N leached (Mg) ---------------------------- | | | | | -----Catchment nitrate-N load (Mg)----- | | | Nitrate-N retained (%) |
| --- | --- | --- | --- | --- | --- | --- | --- | --- | --- | --- |
|  |  | Beef | Dairy | Deer | Sheep | Total | Sum | Lower CI | Upper CI |  |
| AK1 | 268 | 4186 | 10606 | 12 | 1635 | 13790 | 4855 | 3868 | 6172 | 65% |
| AK2 | 83 | 696 | 548 | 18 | 296 | 1631 | 519 | 432 | 629 | 68% |
| AX1 | 4462 | 3888 | 738 | 1433 | 5037 | 11313 | 8779 | 8561 | 8999 | 22% |
| AX2 | 4293 | 2430 | 80 | 430 | 6220 | 9201 | 4832 | 4722 | 4946 | 47% |
| AX3 | 1067 | 249 | 0 | 0 | 969 | 1218 | 798 | 731 | 883 | 34% |
| AX4 | 16586 | 14530 | 4303 | 2615 | 42788 | 65295 | 19896 | 19105 | 20717 | 70% |
| CH1 | 1059 | 848 | 0 | 0 | 1758 | 2606 | 1304 | 1108 | 1530 | 50% |
| CH2 | 2519 | 6931 | 17209 | 436 | 14305 | 39398 | 19750 | 18720 | 20880 | 50% |
| CH3 | 2384 | 1851 | 1582 | 96 | 4508 | 8078 | 9566 | 9200 | 9956 | -18% |
| CH4 | 3014 | 3798 | 19642 | 487 | 8065 | 33146 | 14301 | 13119 | 15603 | 57% |
| DN1 | 2430 | 4588 | 3994 | 97 | 13945 | 22643 | 601 | 510 | 703 | 97% |
| DN2 | 152 | 362 | 0 | 0 | 883 | 1246 | 156 | 54 | 449 | 87% |
| DN3 | 4724 | 10290 | 4258 | 237 | 30856 | 45711 | 3136 | 2419 | 4172 | 93% |
| DN4 | 20569 | 27450 | 36945 | 3682 | 86147 | 156272 | 57919 | 52256 | 64411 | 63% |
| DN5 | 5160 | 11954 | 26331 | 1815 | 49384 | 91275 | 91559 | 88758 | 94441 | 0% |
| DN6 | 785 | 1162 | 11 | 296 | 3882 | 5590 | 4013 | 3912 | 4115 | 28% |
| DN7 | 1133 | 2011 | 3349 | 168 | 4682 | 10212 | 10884 | 10463 | 11322 | -7% |
| DN8 | 2152 | 4600 | 22016 | 1646 | 14172 | 43127 | 40589 | 38589 | 42688 | 6% |
| DN9 | 8132 | 6921 | 7050 | 3104 | 22110 | 39198 | 25276 | 24331 | 26261 | 36% |
| DN10 | 250 | 0 | 0 | 0 | 0 | -^1^ | 144 | 130 | 158 | - |
| GS1 | 1576 | 18478 | 325 | 63 | 13827 | 32913 | 11008 | 8415 | 14579 | 67% |
| GS2 | 27 | 383 | 0 | 7 | 333 | 724 | 316 | 277 | 360 | 56% |
| GS3 | 294 | 4160 | 666 | 463 | 2906 | 8195 | 3718 | 3084 | 4460 | 55% |
| GS4 | 1384 | 8891 | 1581 | 463 | 5220 | 16155 | 8208 | 7191 | 9359 | 49% |
| GY1 | 6302 | 7217 | 41614 | 1291 | 4753 | 54881 | 26066 | 24658 | 27591 | 53% |
| GY2 | 3820 | 8841 | 51269 | 2881 | 3863 | 66855 | 38056 | 36769 | 39380 | 43% |
| GY3 | 641 | 475 | 174 | 220 | 175 | 1045 | 2081 | 1875 | 2309 | -99% |
| GY4 | 1028 | 0 | 0 | 0 | 0 | 0 | 5555 | 5270 | 5856 | - |
| HM1 | 319 | 6005 | 3758 | 6 | 4043 | 13812 | 4378 | 4204 | 4563 | 68% |
| HM2 | 2863 | 32619 | 185714 | 1607 | 16165 | 237989 | 85262 | 82484 | 88067 | 64% |
| HM3 | 8335 | 41942 | 327427 | 4398 | 23189 | 400345 | 61263 | 58191 | 64413 | 85% |
| HM4 | 12373 | 84763 | 590876 | 6111 | 42116 | 729948 | 170064 | 161882 | 178480 | 77% |
| HM5 | 1107 | 3360 | 77180 | 583 | 1244 | 83616 | 41500 | 40977 | 42034 | 50% |
| HM6 | 286 | 1257 | 16228 | 91 | 270 | 17968 | 8401 | 7364 | 9654 | 53% |
| HV1 | 122 | 1588 | 53 | 0 | 1789 | 740 | - | - | - | - |
| HV2 | 2464 | 31560 | 13017 | 1287 | 30572 | 77365 | 37833 | 32634 | 43905 | 51% |
| HV3 | 2009 | 16184 | 3711 | 843 | 14109 | 36352 | 9579 | 7503 | 12286 | 74% |
| HV4 | 386 | 5 | 0 | 0 | 5 | 0 | 374 | 302 | 474 | - |
| HV5 | 2370 | 7747 | 4764 | 1972 | 6185 | 20673 | 15119 | 12969 | 17648 | 27% |
| HV6 | 1038 | 2992 | 4188 | 171 | 3110 | 10460 | 8032 | 7754 | 8315 | 23% |
| NN1 | 1760 | 2856 | 5916 | 166 | 3307 | 12413 | 12408 | 11244 | 13693 | 0% |
| NN2 | 166 | 0 | 0 | 0 | 0 | 0 | 167 | 153 | 182 | - |
| NN3 | 518 | 334 | 0 | 0 | 181 | 516 | 549 | 483 | 627 | -7% |
| NN4 | 3417 | 8030 | 2245 | 572 | 8902 | 20765 | 13614 | 11790 | 15810 | 34% |
| NN5 | 1402 | 2869 | 3057 | 140 | 2280 | 8352 | 2741 | 2556 | 2938 | 67% |
| RO1 | 190 | 699 | 56 | 137 | 339 | 1232 | 10 | 9 | 11 | 99% |
| RO2 | 708 | 2635 | 6649 | 505 | 1023 | 10908 | 10369 | 10254 | 10485 | 5% |
| RO3 | 1171 | 6252 | 10081 | 17 | 5163 | 21528 | 15421 | 15223 | 15617 | 28% |
| RO4 | 510 | 499 | 829 | 5 | 232 | 1564 | 2394 | 2229 | 2584 | -53% |
| RO5 | 2912 | 7833 | 39391 | 531 | 5611 | 53897 | 26975 | 26277 | 27692 | 50% |
| RO6 | 3440 | 15228 | 7984 | 107 | 9559 | 32935 | 191 | 170 | 212 | 99% |
| TK1 | 2370 | 1535 | 2651 | 312 | 3851 | 46623 | 2817 | 2520 | 3185 | 94% |
| TK2 | 411 | 8230 | 15104 | 2287 | 17397 | 9081 | 31611 | 29287 | 34232 | -248% |
| TK3 | 456 | 1106 | 1074 | 104 | 2212 | 4514 | 2272 | 2119 | 2436 | 50% |
| TK4 | 9744 | 6712 | 1086 | 629 | 20590 | 29049 | 3518 | 3217 | 3832 | 88% |
| TK5 | 898 | 8652 | 1933 | 1453 | 25454 | 37903 | 598 | 402 | 906 | 98% |
| TK6 | 11882 | 11067 | 21351 | 1857 | 29914 | 65745 | 57579 | 34689 | 92902 | 12% |
| TU1 | 2190 | 16297 | 6559 | 461 | 17123 | 40476 | 25488 | 23392 | 27715 | 37% |
| TU2 | 788 | 1337 | 771 | 0 | 911 | 3020 | 1336 | 1247 | 1427 | 56% |
| WA1 | 1114 | 8369 | 39958 | 247 | 5023 | 53629 | 21287 | 19763 | 22901 | 60% |
| WA2 | 15 | 84 | 2390 | 0 | 3 | 773 | 393 | 362 | 426 | 49% |
| WA3 | 226 | 455 | 30762 | 2 | 192 | 31438 | 13765 | 13392 | 14140 | 56% |
| WA4 | 6625 | 48384 | 8473 | 865 | 54573 | 112371 | 79803 | 64970 | 99667 | 29% |
| WA5 | 2685 | 21977 | 1435 | 893 | 30330 | 54635 | 8826 | 7529 | 10351 | 84% |
| WA6 | 3444 | 31234 | 9957 | 1131 | 46535 | 88891 | 197260 | 161053 | 237577 | -122% |
| WA7 | 714 | 10007 | 7180 | 379 | 9778 | 27348 | 9382 | 8170 | 10812 | 66% |
| WA8 | 3919 | 38430 | 63922 | 1739 | 44334 | 149149 | 71760 | 66423 | 77622 | 52% |
| WA9 | 4249 | 41756 | 74249 | 2149 | 47688 | 166620 | - | - | - | - |
| WH1 | 122 | 185 | 10 | 0 | 19 | 215 | 255 | 205 | 316 | -19% |
| WH2 | 300 | 5508 | 7864 | 64 | 722 | 14212 | 2948 | 2506 | 3453 | 79% |
| WH3 | 808 | 7236 | 9204 | 0 | 1610 | 18050 | 6761 | 5087 | 9150 | 63% |
| WH4 | 543 | 4802 | 20691 | 1 | 428 | 26134 | 10198 | 8408 | 12256 | 61% |
| WN1 | 605 | 1353 | 1203 | 77 | 859 | 3500 | 5210 | 4973 | 5457 | -49% |
| WN2 | 87 | 162 | 439 | 0 | 70 | 670 | 505 | 475 | 538 | 25% |
| WN3 | 2362 | 16150 | 22232 | 909 | 23150 | 64505 | 34728 | 32491 | 37115 | 46% |
| WN4 | 643 | 5003 | 5572 | 160 | 7407 | 18293 | 12579 | 11871 | 13341 | 31% |
| WN5 | 79 | 102 | 0 | 0 | 36 | 137 | 357 | 335 | 381 | -160% |
|  |  |  |  |  |  |  |  |  |  |  |
| Sum |  | 730,586 | 1,913,484 | 56,925 | 912,323 | 3,642,075 | 1,541,773 | 1,390,065 | 1,724,756 | - |
| Median |  | 4,600 | 4,188 | 237 | 4,682 | 19,483 | 8,779 | 7,529 | 9,654 | 50% |
| Min |  | 0 | 0 | 0 | 0 | 0 | 10 | 9 | 11 | -248% |
| Max |  | 84,763 | 590,876 | 6,111 | 86,147 | 729,948 | 197,260 | 161,882 | 237,577 | 99% |
| Skewness | | 2.91 | 5.85 | 2.43 | 2.07 | 5.11 | 3.27 | 3.14 | 3.39 | -2.55 |

^1^ Refers to either a load or percentage retention that could not be calculated.

**Pre-whitening the data for analysis by cross-correlation**

We undertook to pre-whiten time-series before calculating the cross-correlation function^1^ despite some calls against this^2^. This ensures that potential autocorrelation and non-stationarity within the two series do not falsely produce significant CCF. When pre-whitened data are used, both the frequency of detecting a significant lag time and the mean lag time decreases (Fig. S1). We were more confident of the pre-whitened data than the raw data because raw data indicated lag times were often less than the mean transit time of baseflow, which is the theoretical minimum hydrologic lag time. For example, mean raw cross-correlation lag times for WA4 (6 years), WA5 (3.5 years), and WA6 (<1 years) were at or potentially lower than the measured mean transit times at these sites of 6-10, 3-6, and 3-6 years, respectively. Pre-whitened data were not – as they could not resolve a lag time (see Table 2).

**Fig S1**. Range and summary statistics of the lag time between nitrate leaching and load in the river across all 76 sites using the raw and pre-whitened data. Boxes describe the 25, 50 and 75^th^ percentiles and whiskers the 5 and 95^th^ percentiles. Outliers and indicated by black dots.


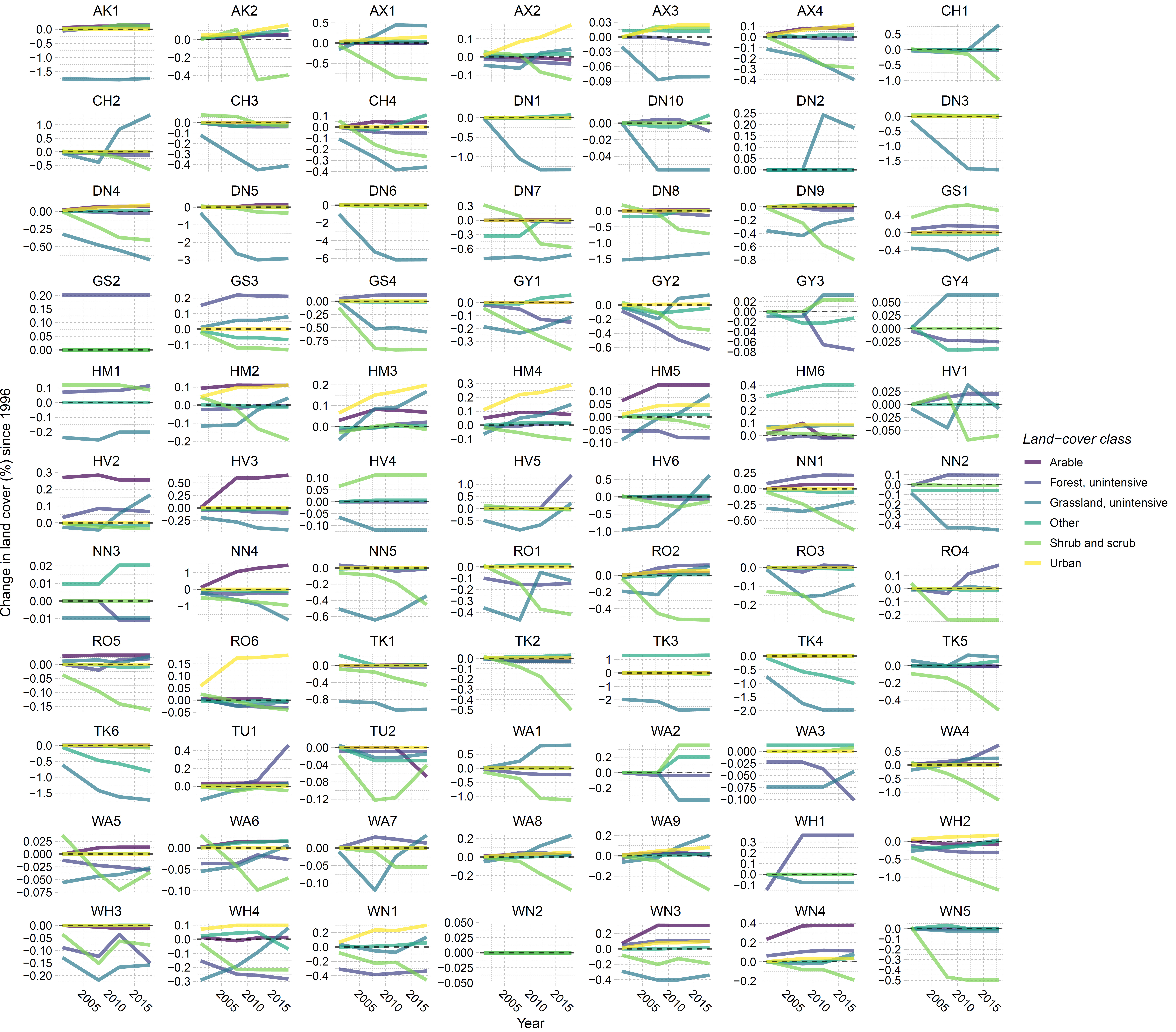


**Fig S2**. Percentage change in arable, native forest, extensive grassland, scrub, urban and other land uses in each of the catchments in 2018 relative to the 1996 Agricultural Census .


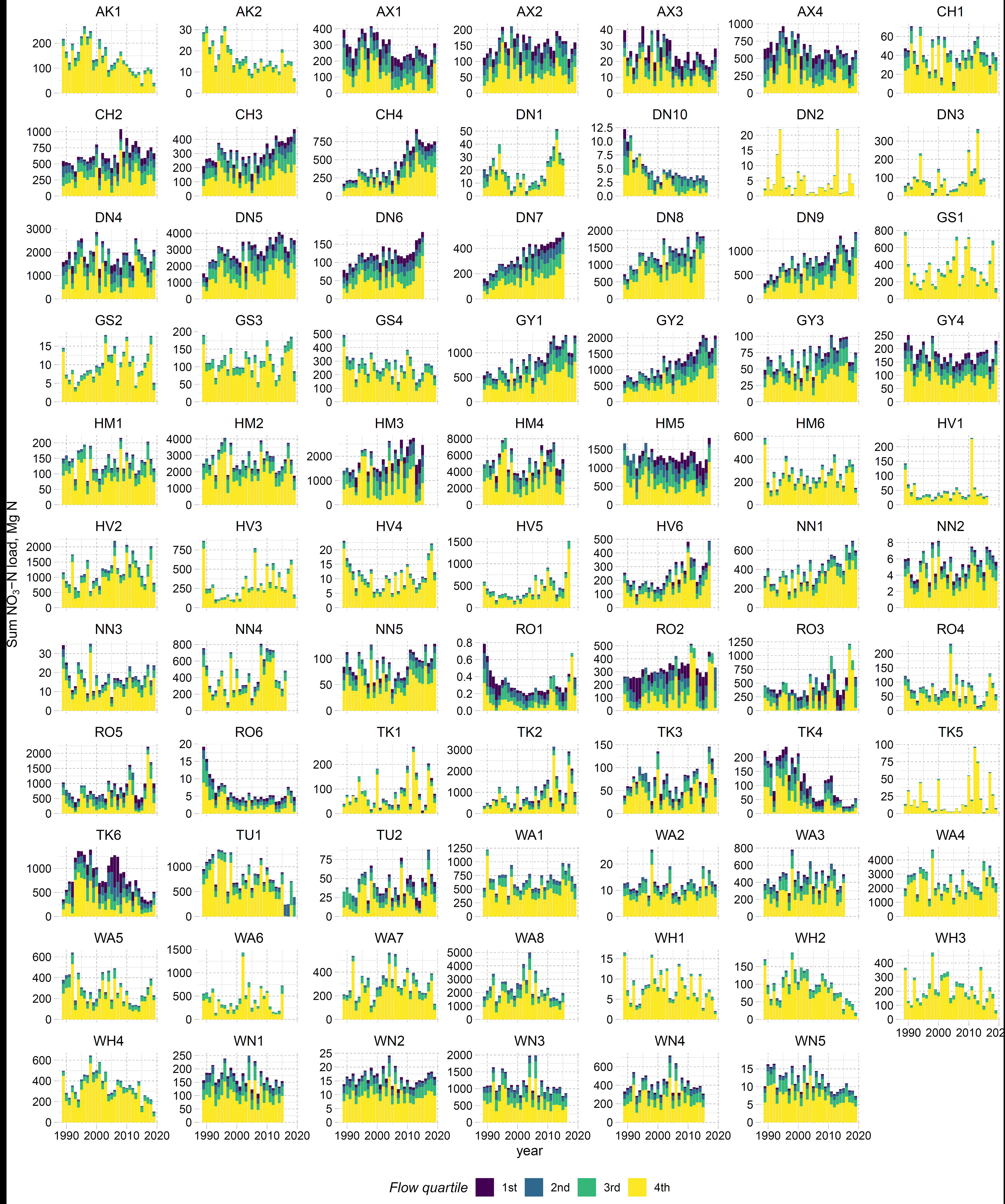


**Fig. S3**. Nitrate-N loads attributable to different flow quartiles (calculated across entire record) showing the disproportionate contribution of high flows (Q4) to annual nitrate-N loads for a selection of rivers from the National River Water Quality Monitoring Network.

**Table S2**. Output of a best subsets regression to predict mean lag time from catchment variables.

| Variables | R^2^ | R^2^ (adj) | Mallows Cp | S | AIC | Area (km^2^) | Mean slope (degrees) | Mean altitude (MSL) | Mean runoff (mm) | Evapotranspiration (mm) | Population density (people km^-2^) | Geology class | % Forest | Olsen P( mg kg^-1^) | % Urban | Stream order | Baseflow index | % Intensive agricultural land |
| --- | --- | --- | --- | --- | --- | --- | --- | --- | --- | --- | --- | --- | --- | --- | --- | --- | --- | --- |
| 1 | 11.5 | 8.5 | 19.7 | 2.07 | 142 |  |  |  |  |  |  |  |  |  |  | X |  |  |
| 1 | 10.0 | 7.0 | 20.5 | 2.09 | 142 |  |  |  |  |  |  | X |  |  |  |  |  |  |
| 2 | 18.8 | 13.2 | 17.8 | 2.02 | 142 |  |  |  |  |  |  | X |  |  |  | X |  |  |
| 2 | 17.3 | 11.6 | 18.6 | 2.04 | 142 |  | X | X |  |  |  |  |  |  |  |  |  |  |
| 3 | 30.2 | 22.7 | 13.7 | 1.90 | 140 |  | X | X |  |  |  |  |  |  |  | X |  |  |
| 3 | 30.0 | 22.5 | 13.8 | 1.91 | 140 |  | X | X |  |  |  | X |  |  |  |  |  |  |
| 4 | 42.7 | 34.2 | 8.9 | 1.76 | 136 |  | X | X |  | X |  |  |  |  |  | X |  |  |
| 4 | 42.4 | 33.9 | 9.1 | 1.76 | 137 |  | X | X |  |  |  |  |  | X |  | X |  |  |
| 5 | 52.9 | 43.8 | 5.4 | 1.62 | 133 |  | X | X |  | X |  | X |  |  |  | X |  |  |
| 5 | 50.3 | 40.8 | 6.8 | 1.67 | 135 |  | X | X |  | X |  |  | X |  |  | X |  |  |
| 6 | 57.5 | 47.3 | 4.9 | 1.57 | 134 |  | X | X |  | X | X | X |  |  |  | X |  |  |
| 6 | 56.9 | 46.6 | 5.2 | 1.58 | 134 |  | X | X |  | X | X |  | X |  |  | X |  |  |
| 7 | 60.9 | 49.6 | 5.1 | 1.54 | 135 |  | X | X |  |  | X | X |  | X |  | X | X |  |
| 7 | 60.6 | 49.0 | 5.3 | 1.54 | 135 |  | X | X |  | X | X | X |  |  |  | X | X |  |
| 8 | 64.4 | 52.0 | 5.2 | 1.50 | 136 |  | X | X |  | X | X | X |  | X |  | X | X |  |
| 8 | 62.9 | 49.9 | 6.0 | 1.53 | 138 |  | X | X |  | X | X | X | X |  |  | X | X |  |
| 9 | 65.5 | 51.4 | 6.6 | 1.51 | 140 |  | X | X |  | X | X | X | X | X |  | X | X |  |
| 9 | 65.1 | 50.9 | 6.8 | 1.52 | 140 | X | X | X |  | X | X | X |  | X |  | X | X |  |
| 10 | 66.0 | 49.8 | 8.3 | 1.53 | 145 | X | X | X |  | X | X | X | X | X |  | X | X |  |
| 10 | 65.6 | 49.2 | 8.5 | 1.54 | 145 | X | X | X |  | X | X | X |  | X |  | X | X | X |
| 11 | 66.3 | 47.8 | 10.2 | 1.56 | 150 | X | X | X |  | X | X | X | X | X | X | X | X |  |
| 11 | 66.1 | 47.5 | 10.3 | 1.57 | 150 | X | X | X | X | X | X | X | X | X |  | X | X |  |
| 12 | 66.5 | 45.4 | 12.1 | 1.60 | 157 | X | X | X | X | X | X | X | X | X | X | X | X |  |
| 12 | 66.3 | 45.0 | 12.2 | 1.60 | 157 | X | X | X |  | X | X | X | X | X | X | X | X | X |
| 13 | 66.6 | 42.5 | 14.0 | 1.64 | 164 | X | X | X | X | X | X | X | X | X | X | X | X | X |

**References**

1 Cryer, J. D. & Chan, K.-S. *Time Series Analysis with Applications in R*. 2nd Edition edn, (Springer-Verlag, 2008).

2 Van Meter, K. J. & Basu, N. B. Time lags in watershed-scale nutrient transport: an exploration of dominant controls. *Environmental Research Letters* **12**, 084017 (2017).
